# Efficacy of Endoscopic Ultrasound-Guided Ablation with the HybridTherm Probe in Locally Advanced or Borderline Resectable Pancreatic Cancer: A Phase II Randomized Controlled Trial

**DOI:** 10.3390/cancers13184512

**Published:** 2021-09-08

**Authors:** Sabrina Gloria Giulia Testoni, Maria Chiara Petrone, Michele Reni, Gemma Rossi, Maurizio Barbera, Valeria Nicoletti, Simone Gusmini, Gianpaolo Balzano, Walter Linzenbold, Markus Enderle, Emanuel Della-Torre, Francesco De Cobelli, Claudio Doglioni, Massimo Falconi, Gabriele Capurso, Paolo Giorgio Arcidiacono

**Affiliations:** 1 Pancreas Translational & Clinical Research Center, Pancreato-Biliary Endoscopy & Endosonography Division, San Raffaele Scientific Institute IRCCS, Vita-Salute San Raffaele University, 20132 Milan, Italy; testoni.sabrinagloriagiulia@hsr.it (S.G.G.T.); petrone.mariachiara@hsr.it (M.C.P.); rossi.gemma@hsr.it (G.R.); capurso.gabriele@hsr.it (G.C.); 2 Pancreas Translational & Clinical Research Center, Oncology Department, San Raffaele Scientific Institute IRCCS, 20132 Milan, Italy; reni.michele@hsr.it; 3 Pancreas Translational & Clinical Research Center, Department of Radiology & Center for Experimental Imaging, San Raffaele Scientific Institute IRCCS, Vita-Salute San Raffaele University, 20132 Milan, Italy; barbera.maurizio@hsr.it (M.B.); nicoletti.valeria@hsr.it (V.N.); gusmini.simone@hsr.it (S.G.); decobelli.francesco@hsr.it (F.D.C.); 4 Pancreas Translational & Clinical Research Center, Pancreatic Surgery Department, San Raffaele Scientific Institute IRCCS, Vita-Salute San Raffaele University, 20132 Milan, Italy; balzano.gianpaolo@hsr.it (G.B.); falconi.massimo@hsr.it (M.F.); 5 ERBE Research Elektromedizin GmbH, 72072 Tübingen, Germany; Walter.Linzenbold@erbe-med.com (W.L.); Markus.Enderle@erbe-med.com (M.E.); 6 Unit of Immunology, Rheumatology, Allergy and Rare Diseases, Pancreas Translational & Clinical Research Center, San Raffaele Scientific Institute IRCCS, Vita-Salute San Raffaele University, 20132 Milan, Italy; dellatorre.emanuel@hsr.it; 7 Pancreas Translational & Clinical Research Center, Pathology Department, San Raffaele Scientific Institute IRCCS, Vita-Salute San Raffaele University, 20132 Milan, Italy; doglioni.claudio@hsr.it

**Keywords:** pancreatic cancer, ablation technique, endoscopic ultrasonography, randomized controlled trial

## Abstract

**Simple Summary:**

Recently, tumour local thermal ablation has been investigated in pancreatic ductal adenocarcinoma (PDAC), hypothesizing that it may add local efficacy to the chemotherapy systemic activity inducing changes of tumour microenvironment and increased intra-tumour drug efficacy. In this phase II randomized controlled trial we investigated the efficacy of thermal ablation with the HybridTherm probe under endoscopic ultrasound-guidance (EUS) as complement to chemotherapy (HTP-CT arm) versus standard chemotherapy alone (CT arm), in locally advanced and borderline resectable PDAC. A sample size of 33 patients per arm was calculated to verify a 20% improved 6-months progression-free survival (6-PFS) rate with HTP-CT. We randomized 17 and 20 patients to HTP-CT and CT arms, respectively. Although not significantly, we found an improved 6-PFS rate in the HTP-CT arm. However, the overall survival was similar between the two arms. Thus, as the study is underpowered, further investigation of EUS-guided thermal ablation in selected patients is suggested.

**Abstract:**

Endoscopic ultrasound-ablation with HybridTherm-Probe (EUS-HTP) significantly reduces tumour volume (TV) in locally-advanced pancreatic ductal adenocarcinoma (LA-PDAC). We aimed at investigating the clinical efficacy of EUS-HTP plus chemotherapy versus chemotherapy (HTP-CT and CT arms) in LA- and borderline-resectable (BR) PDAC, with 6-months progression-free survival (6-PFS) rate as primary endpoint. In a phase-II randomized-controlled-trial, 33 LA/BR-PDAC patients per-arm were planned to verify 20% improved 6-PFS rate. Radiological response (Choi criteria), TV and serum CA19.9 were assessed up to 6-months. Seventeen and 20 LA/BR-PDAC patients were randomized to HTP-CT or CT. Baseline and CT-related features were balanced. At 6-months, 6-PFS rate was 41.2% and 30% in HTP-CT and CT arms (*p* = 0.48), respectively. A decrease ≥50% of serum CA19.9 was achieved in 75% and 64.3% of HTP-CT and CT patients (*p* = 0.53), respectively. TV reduced up to 6-months in 64.3% and 47.1% of HTP-CT and CT patients (*p* = 0.35), respectively. Resection rate, PFS-time and overall survival (OS-time) were similar. HTP-CT achieves a non-significant 11.2%, 10.7% and 17.2% improved 6-PFS, CA19.9 decrease ≥50% and TV reduction rates over CT, without any impact on resection rate, PFS-time and OS-time. As the study was underpowered, these results suggest further investigation of EUS-local ablation in selected patients with localized disease after induction CT.

## 1. Introduction

The National Comprehensive Cancer Network (NCCN) subclassified the “locally-advanced” pancreatic ductal adenocarcinoma (PDAC) into borderline-resectable (BR) and locally-advanced (LA) categories and established the 4- or 6-months induction chemotherapy (CT) as standard of care to control and eventually downsize the LA-PDAC and select the BR-PDAC at higher risk of non-radical resection [1,2]. However, CT and radiotherapy regimens in LA-PDAC have only led to a marginal survival improvement [2,3,4,5].

Local thermal ablation (LTA) has been investigated for LA-PDAC [6], hypothesizing that it may add local efficacy to the CT systemic activity, possibly leading to changes of the desmoplastic PDAC microenvironment and increased intra-tumour drug efficacy [7]. Their application in PDAC has been limited because of thermal injury risk to the pancreas and surrounding structures.

We have reported that ablation with the HybridTherm-Probe (HTP, ERBE ElektroMedizin GmbH, Tübingen, Germany) [8,9,10], an internally carbon dioxide–cooled bipolar radiofrequency (RF)-energy device, under endoscopic ultrasound (EUS)-guidance, was safe in 22 LA-PDAC patients after failure of first-line CT or unfit for CT, inducing a significant tumour volume reduction at 1-month imaging. We also showed that the tumour volume reduction rate and radiological response using the Choi criteria were significantly associated with the patients’ overall survival (OS) [10].

No randomized clinical trials (RCTs) have investigated whether the addition of LTA to CT results in improved clinical outcomes in PDAC. The present single center phase II RCT aimed to explore the EUS-HTP efficacy in controlling the tumour progression of LA/BR-PDAC, comparing the effects of upfront EUS-HTP plus CT versus CT alone.

## 2. Materials and Methods

This RCT (N. HTP/2014; NCT02336672) was approved by the Ethics Committee of the San Raffaele Scientific Institute (Milan, Italy) and performed according to the Declaration of Helsinki guidelines. Written informed consent was obtained from each patient for enrolment, treatments and data collection.

### 2.1. Study Population and Design

Inclusion criteria were: age 18–80 years; PDAC cytological/histological diagnosis and radiological LA/BR staging (NCCN criteria [1], Union for International Cancer Control (UICC) Tumour-Node-Metastasis classification [11]), by contrast-enhanced (CE) total-body multi-detector computed-tomography (MDCT), abdomen double-weighed magnetic resonance imaging (DW-MRI), EUS and eventually positron emission tomography (PET); patients treatment naïve and candidate to CT; lesion size ≥ 30 mm; Karnofsky Performance Status ≥ 70%; life expectancy > 6 months; white blood count ≥ 3500/mm^3^, neutrophils ≥ 1500/mm^3^, platelets > 100,000/mm^3^, haemoglobin ≥ 10 g/dL, creatinine ≤ 1.5 mg/dL, International Normalized Ratio (INR) < 1.5.

Eligible patients, identified by a multidisciplinary team with PDAC expertise, were subsequently randomly allocated (1:1) within 3 weeks from basal staging to receive upfront EUS-HTP plus CT (HTP-CT arm) or CT alone (CT arm). Randomisation was performed using a computer-generated random list generator held by ERBE, responsible also for monitoring data accuracy, completeness and reliability. Investigators and patients were not masked to treatment allocation. An internal research team was responsible for data collection in case record forms.

In the HTP-CT arm, up to 3 EUS-HTP sessions were planned, at one month apart each other, based on the tumour’s size allowing HTP insertion into lesion, and patient’s conditions, in absence of PD. CT was started after one week from the first EUS-HTP session.

In both the arms, CT was planned at the oncologists’ discretion according to the Medical Oncology Italian Association (AIOM) guidelines (Table S1), to ensure adherence to the oncological therapy, and administered for a minimum of 6 cycles, or until progressive disease (PD), unacceptable toxicity, patient’s refusal or medical decision.

Restaging evaluations were performed at 2-, 4- and 6-months from CT onset at San Raffaele Scientific Institute, using CE-MDCT, DW-MRI and, if necessary, PET, and measuring the carbohydrate-antigen 19.9 (CA19.9) serum levels. 

At 4- and 6-months, in absence of radiological or biological PD, patients were evaluated for surgical exploration. Patients who at 4-months showed unresectable tumour, without metastases, prosecuted on CT for other 2 months. At 6-months, patients who were unsuitable for resection and still PD-free prosecuted on concomitant chemoradiotherapy.

Chemoradiotherapy was also recommended as adjuvant therapy (Figure S1).

The end of the trial was the date of the 6-months evaluation of the last enrolled patient. All patients were followed-up until death. Database lock for the present analysis was February 2020, when all patients had completed a minimum of 6-months follow-up.

### 2.2. Study Methods

As previously described [8,9,10], the active tip (26-mm length) of the needle-shaped (14-gauge) HTP was placed directly into target lesion under EUS-guidance using colour power-doppler to avoid vascular structures, and activated at fixed RF power of 18W and cooling pressure of 650 psi, with application time between 240 and 480 s for a 2-cm up to >3-cm mass or until the electric resistance, induced by tumour tissue desiccation and devitalization, increased.

Serum blood count with leukocyte formula, amylase, lipase, activated-protein-C, lactate-dehydrogenase, glucose, calcium, creatinine, INR and CA19.9 were assessed at the post-operative 3-days, along with CE-MDCT and DW-MRI to exclude adverse events (AEs). Timing and severity of EUS-HTP related AEs were classified according to the American Society for Gastrointestinal Endoscopy (ASGE) lexicon for endoscopic AEs [12]. 

In both the arms, blood tests were performed before treatments and repeated after 2-, 4- and 6-months, in conditions of jaundice absence for CA19.9 measurements.

In patients with CA19.9 > 34 U/mL (upper normal limit), the lowest value measured at any time for each patient compared with baseline represented the CA19.9 nadir. Patients with <50%, 50–89%, and ≥90% decrease of CA19.9 at nadir were defined as biological non-, minor- and major-responders [13].

Radiological response to treatment was determined according to RECIST1.1 and revised using Choi-criteria [14,15], which consider changes of tumour size and density attenuation coefficient, evaluating the difference of imaging assessments between the previous and current examinations. We used Choi-criteria after recently confirming that they allowed better prediction of OS than RECIST1.1 in LA-PDAC patients treated with EUS-HTP after primary CT [11]. Patients with complete response, partial response (PR) or stable disease (SD) to treatment were defined as presenting disease control; those experiencing PD were non-responders.

Tumour volume (cubic centimeter, cc) was also measured at each follow-up, using a computer-aided detection system (IntelliSpace Portal-ISP7.0, Philips Medical System DMC, Hamburg, Germany) [10,16]. Post-HTP vital tumour volume was defined as the difference between the lesion and necrosis volumes [10,17,18]. These measurements were double-checked by two radiology experts in pancreatic imaging.

### 2.3. Study Outcomes

Primary endpoint was the 6-months progression-free survival (6-PFS) rate, evaluated as the proportion of patients still PD-free at 6-months follow-up.

Secondary endpoints were: (a) proportion of patients who achieved radiological and biological response to treatment; (b) post-treatment vital tumour volume reduction rate, calculated as the vital tumour volume percentage variation between the baseline and current examinations; (c) radical (R0) resection rate after 4-/6-months CT; (d) EUS-HTP-related feasibility and complication rates; (e) PFS- and OS-time, evaluated as the time interval from the randomisation to the first radiological evidence of PD (or death) and to the death or last follow-up assessment.

### 2.4. Sample Size Calculation and Statistical Analysis

A 20% increase of the 6-PFS rate was hypothesized when using HTP-CT versus CT (80% vs. 60%), based on previous reports showing that 6-PFS rate ranged at around 50% with Gemcitabine and 70% with PEXG [19,20]. With 5% significance level, 80% power and 10% estimated drop-out, the final sample calculation was of 100 patients per arm. This RCT, originally designed as multi-center, consisted of two phases (II/III), with the first one including 33 patients per arm to assess whether the sample of patients who receive HTP-CT shows a 6-PFS improvement over CT alone. According with the power calculation, if the 6-PFS difference between the two arms in the phase II trial was: (a) ≥20% the trial could go on; (b) between 5–19% the trial could go on with a sample size recalculation; (c) <5% the trial had to be stopped. This study presents the results from the phase II trial. Student t-test (mean ± standard deviation, StDe) or Mann-Whitney and Wilcoxon correction tests (median with interquartile range (IQR) for continuous variables, accordingly to the Shapiro-Wilk test for normal distribution, and Fisher’s exact test for categorical variables (numbers and percentages), are used to assess inter-arm differences. Safety analysis is performed on patients receiving at least one EUS-HTP session and/or one cycle of CT. The Kaplan-Meier survival curves are compared using the Log-rank test (Chi-squared statistic). The median progression-free survival and overall survival time and their 95% confidence interval (CI) are calculated according to Brookmeyer & Crowley, 1982 [21]. The hazard ratios and their 95% CI are calculated according to Altman et al., 2000 [22]. Data analysis is performed both as Intention-To-Treat (ITT–set), including all randomized patients meeting eligibility criteria, and Per-Protocol (PP-set), including patients who did not violate the protocol, using MedCalc version 19.2.1 (MedCalc Statistical Software bvba, Ostend, Belgium). *p*-values ≤ 0.05 are considered as statistically significant.

## 3. Results

The study was redesigned as single centre (San Raffaele Scientific Institute) due to difficulties of other centres to enrol and follow-up patients. Between November 2014 and June 2019, 40 patients were randomly allocated to HTP-CT arm (*n* = 20) or CT arm (*n* = 20). Due to the slow accrual due to inclusion criteria and the HTP withdrawal by the manufacturer in 2020, it was established to stop enrolment and perform the phase II trial analysis despite the smaller sample size than the required.

### 3.1. Patients and Therapeutic Features

**Figure 1:**
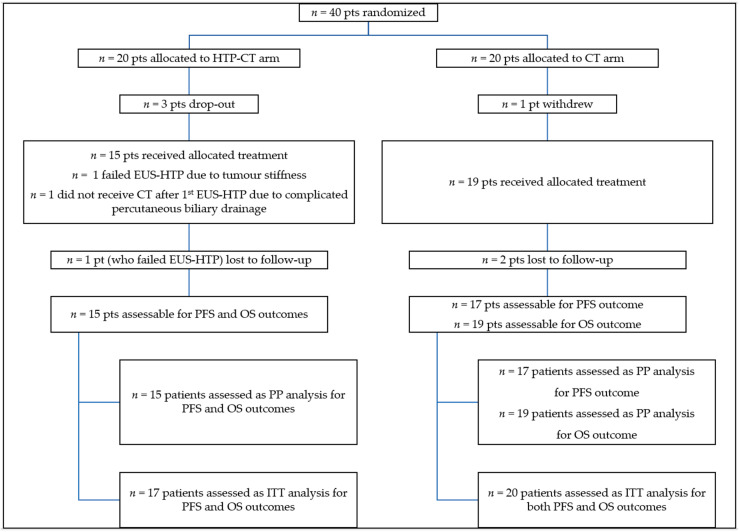
Patients CONSORT flow-diagram. Pts: patients; N: number; HTP-CT: HybridTherm Probe plus Chemotherapy; CT: Chemotherapy; EUS-HTP: endoscopic ultrasound-guided ablation with HybridTherm Probe; PFS: progression-free survival; OS: overall survival; PP: per-protocol; ITT: intention-to-treat.

Figure 1 shows the flow-diagram of enrolled patients. 

Three patients of the HTP-CT arm were excluded after randomisation because metastatic disease was detected at MDCT/MRI re-evaluation before EUS-HTP.

In the HTP-CT arm, 7 patients were treated with three HTP sessions, other 7 with two sessions and 2 with one session. In one patient EUS-HTP was not feasible due to tumour stiffness. Thus, EUS-HTP feasibility rate was 94.1% (mean application time: 175.3 ± 39.6 s; mean ablation size: 21.9 ± 5.1 mm × 30.3 ± 5.9 mm). One HTP-CT patient did not receive CT after EUS-HTP due to complicated percutaneous biliary drainage. Assigned HTP-CT treatment was, therefore, performed in 15 patients. Second and third sessions were feasible in 14/15 and 7/11 eligible patients. In 5 patients (one at the second session), EUS-HTP was not performed because of tumour reduction (≤26 mm). Other patients were not eligible to third session because of PD.

In the CT arm, one patient withdrew after randomization. Other 19 patients received the allocated treatment. Two patients were lost to follow-up (after 52 and 73 days) due to patients’ choice. 

CT-related features were evenly balanced between the two arms (Table S2). 

The PFS-time and OS-time were assessable both in 15 patients (17 ITT-set) of the HTP-CT arm and, respectively, in 17 and 19 patients (20 ITT-set) of the CT arm.

Statistically, baseline clinical and tumour features of HTP-CT (*n* = 17) and CT (*n* = 20) patients were evenly balanced (Table 1).

### 3.2. Six-Months Progression-Free Survival and Radiological Response to Therapy

Using Choi criteria, the 6-months PFS rate was of 41.2% and 30% in the HTP-CT and CT arms, respectively, with 7/17 HTP-CT and 6/20 CT patients still PD-free (*p* = 0.48). Table 2 reports the radiological response using Choi criteria and RECIST1.1.

In the HTP-CT and CT arms, respectively, partial response was achieved in 9/17 (52.9%) and 11/20 (55%) patients, whilst stable disease in 3/17 (17.6%) and 3/20 (15%) patients. 

The overall radiological response rate was of 71% (12/17) and 65% (13/20) in the HTP-CT and CT arms, respectively (*p* = 0.95).

Two-months PD occurred in 17.6% and 20% (RECIST1.1:17.6% and 15%) of HTP-CT and CT patients, respectively.

Local PD developed in 23.5% and 40% (RECIST1.1:11.8% and 35%) whilst distant PD in 29.4% and 25% of HTP-CT and CT patients, respectively.

### 3.3. Biological Response to Therapy

Compared with baseline, minor and major biological responses were, respectively, recorded in 9/16 (56.3%) and 3/16 (18.8%) HTP-CT patients, whilst in 5/14 (35.7%) and 4/14 (28.6%) CT patients. 

The overall biological response rate was of 75% (12/16) and 64.3% (9/14) in the HTP-CT and CT arms (*p* = 0.53), respectively (Table 3).

Among the non-responders, a decrease < 50% was recorded in 3/14 CT patients (21.4%), and an increase was observed in the other patients (*n* = 2 of the HTP-CT arm, *n* = 1 of the CT arm).

The median time to CA19.9 nadir was 4 months (IQR: 2–5) and 3.5 months (IQR: 2–4) in the HTP-CT and CT arms, respectively.

### 3.4. Tumour Volume Changes

Three and two patients of HTP-CT and CT arms, respectively, refused to undergo DW-MRI and had the tumour volume measured on CE-MDCT [13]. 

In the HTP-CT and CT arms, respectively, the vital tumour volume was measured in 14/15 and 17/17, 12/14 and 16/17, 10/12 and 12/13 patients at 2-, 4- and 6-months. In the remaining patients the tumour and/or necrotic area had unclear edges that did not allow calculation.

Overall, 10/14 (71.4%) and 13/17 (76.5%) patients of the HTP-CT and CT arms, respectively, experienced a vital tumour volume reduction compared with baseline, with a significant difference between the two treatment arms at 2-months (*p* = 0.02). However, observing the vital tumour volume trend during the follow-up, 9/14 (64.3%) and 8/17 (47.1%) patients of the HTP-CT and CT arms, respectively, showed a consistent vital tumour volume reduction rate up to 6-months, without a significant difference (*p* = 0.39); in the other patients it increased over time. Compared with baseline, a maximum median vital tumour volume reduction rate of 43.7% (IQR: −64.8–27.7%) and 22.1% (IQR: −31–43.9%) was recorded in the HTP-CT and CT arms, respectively, at 6-months (Table 4), even if not significantly in both the treatment arms.

### 3.5. Surgical Outcomes

Two HTP-CT patients (*n* = 1 LA, *n* = 1 BR) were resected after 6 cycles of induction CT and two EUS-HTP sessions with tumour reduction.

In the CT arm, one (LA) and other 3 patients (*n* = 2 LA, *n* = 1 BR) were resected after 5 and 6 cycles of induction CT, respectively, despite the best response was SD in all of them.

The achieved resection rate was of 11.8% (2/17) and 20% (4/20) in the HTP-CT and CT arms, respectively (*p* = 0.51), with R0 resection in both the HTP-CT patients and in 3/4 CT patients. 

Pathological results [11,23] are reported in Table S3.

### 3.6. Adverse Events

AEs occurred in 11/37 EUS-HTP procedures (29.7%). Of these, 8.1%, 10.8% and 10.8% were intra-procedural (*n* = 1 moderate and *n* = 2 mild bleeding at needle puncture site resolved, respectively, through endoscopic intervention and spontaneously), early (*n* = 1 moderate jaundice requiring endoscopic biliary stenting, *n* = 3 mild including fever, splenic vein thrombosis and asymptomatic perigastric collection, resolved with medical therapy or spontaneously) and potentially late (*n* = 3 moderate jaundice requiring endoscopic biliary stenting, *n* = 1 mild duodenal ulcer at needle puncture site resolved with medical therapy). 

No signs of acute pancreatitis were observed at 72-h imaging and laboratory control, well as no severe AEs occurred. 

EUS-HTP was safe in all patients carrying biliary metal stents.

The rates of grade III/IV CT-related AEs (National Cancer Institute’s Common Terminology Criteria for Adverse Events, version 4.0) [24] were similar between the two arms (Table S4).

### 3.7. Progression-Free and Overall Survival

The median PFS-time (Choi criteria) was 11.1 (95% CI: 4.0–40.6) and 9.4 (95% CI: 4.1–23.1) months in the HTP-CT and CT arms, respectively. At Kaplan-Meier survival curves analysis the HR CT arm/HTP-CT arm for progression was of 1.16 (95% CI: 0.6–2.3; Log-rank test: Chi-squared = 0.17, *p* = 0.68). 

**Figure 2:**
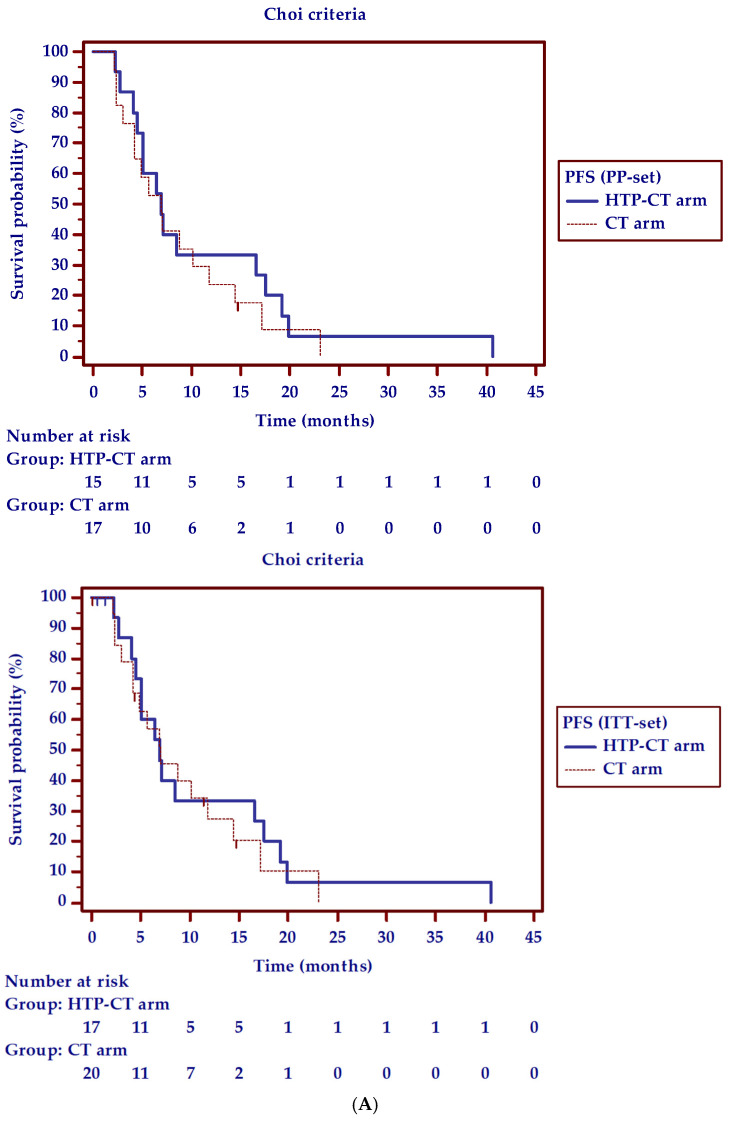

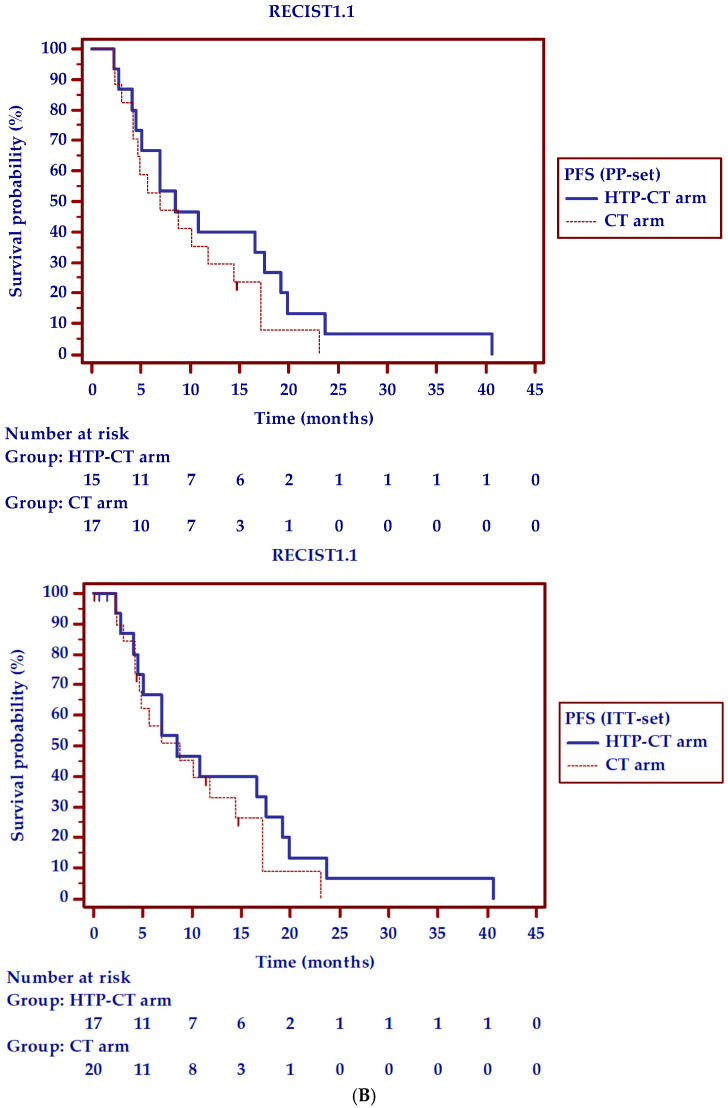
Median progression-free survival (PFS) time (95% confidence interval) and hazard ratio (HR, 95% confidence interval) for progression in patients treated with endoscopic ultrasound-guided ablation with HybridTherm Probe plus chemotherapy (HTP-CT arm) versus chemotherapy alone (CT arm), according to: (**A**) Choi criteria. Per-Protocol (PP-set): 11.1 (4.0– 0.6) versus 8.7 (3.0–23.1) months; HR CT arm/HTP-CT arm for progression = 1.26 (0.6–2.6) (Log-rank test: Chi-squared = 0.41; *p* = 0.52); Intention-To-Treat (ITT-set): 11.1 (4.0–40.6) versus 9.4 (4.1–23.1) months; HR CT arm/HTP-CT arm for progression = 1.16 (0.6–2.3) (Log-rank test: Chi-squared = 0.17; *p* = 0.68). (**B**) RECIST1.1. Per-Protocol (PP-set): 12.6 (4.0–40.6) versus 9.6 (4.1–23.1) months; HR CT arm/HTP-CT arm for progression = 1.36 (0.7–2.8) (Log-rank test: Chi-squared = 0.72; *p* = 0.39); Intention-To-Treat (ITT-set): 12.6 (4.0–40.6) versus 10.2 (4.2–23.1) months; HR CT arm/HTP-CT arm for progression = 1.26 (0.6–2.6) (Log-rank test: Chi-squared = 0.41; *p* = 0.52). Short vertical lines represent censured patients.

Figure 2 shows the PFS curves using both Choi criteria and RECIST1.1.

Overall median follow-up was of 45.4 months (95% CI: 45.3–45.4). At 18-month follow-up, 7/17 (41.2%) and 9/20 (45%) patients of the HTP-CT and CT arms, respectively, were still alive. 

The median OS-time was 13 (95% CI: 8.8–25.5) and 17 (95% CI: 10.9–56.7) months in the HTP-CT and CT arms, respectively. At Kaplan-Meier survival curves analysis the HR HTP-CT arm/CT arm for death was of 1.18 (95% CI: 0.6–2.4; Log-rank test: Chi-squared = 0.20, *p* = 0.79; Figure S2).

Excluding 3 HTP-CT and 4 CT patients with 2-months distant PD, the median PFS- (Choi criteria) and OS-time were, respectively, 10.1 (95% CI: 5.0–40.6) and 20.5 (95% CI: 10.5–30.2) months in the HTP-CT arm and 7.1 (95% CI: 4.9–23.1) and 18.5 (95% CI: 10.1–56.7) months in the CT arm, with a HR CT arm/HTP-CT arm for progression of 1.18 (95% CI: 0.5–2.6; Log-rank test: Chi-squared = 0.16, *p* = 0.69) and for death of 1.2 (95% CI: 0.5–2.5; Log-rank test: Chi-squared = 0.12, *p* = 0.79).

## 4. Discussion

By inducing intra-tumour coagulative necrosis, LTA would be of clinical relevance during neoadjuvant treatment within a multidisciplinary approach in LA/BR PDAC. However, the potential clinical benefit of LTA added to CT versus CT alone in LA/BR-PDAC was not yet evaluated in RCTs [25].

This phase II RCT did not reach the primary endpoint of better PFS in patients treated with combined HTP-CT. 

The study was originally designed to be multi-centric, including 33 patients per arm, to assess the 6-months rate of patients still PD-free, that seems to be a more reliable measure of the treatment activity than others for deciding to test a PDAC treatment in a phase III trial [26].

Unfortunately, due to unforeseen problems in patients’ enrolment in other centres and HTP withdrawal by the manufacturer as consequence, the study was redesigned as single-centre including only 17 patients of the HTP-CT arm and 20 patients of the CT arm. Thus, it is underpowered to allow solid conclusions about the difference in the 6-PFS rate between the HTP-CT and CT treatments. Nevertheless, it provides helpful insights in the concept of combined CT and LTA.

The statistically balanced baseline features between the two arms and the survival outcome of the control arm similar to those seen with the same CT regimens in recent reports in LA/BR-PDAC [27] do not suggest major patient selection bias.

Few studies have dealt with EUS-LTA. Most published reports described the percutaneous image-guided and laparotomic routes, with a 20% major complication rate and 25% mortality rate after intra-operative RFA, as well as major and minor complication rates of 10% and >50% after cryosurgery [25].

This study confirmed the EUS-HTP safety in PDAC [9,10]. With respect to the other similar monopolar devices performing EUS-guided RFA, despite the less handy 14-gauge diameter the HTP device takes advantage from the bipolar and cooling systems to create larger ablation areas with less power input and less collateral thermal damage than other available EUS-RFA probes. 

On the other hand, the development of probes with smaller gauges as well as of different RFA devices with different tip length according to the tumour size would overcome the limit of unique HTP application to lesion ≥ 30 mm. Three small sample phase I studies evaluated the feasibility and safety of EUS-guided RFA, in unresectable non-metastatic PDAC mainly not suitable for additional CT, with the monopolar 19-gauge EUSRA probe (5-, 7-, 10-, 15-, 20-, 25- and 30-mm electrode) from Taewoong Medical (Taewoong Medical Co., Ltd., Gimpo-si, Gyeonggi-do, South Korea), reporting an AEs rate of 37.5%, with only mild AEs [28,29,30]. Other two studies (11 and 8 patients, respectively) applied the 19-gauge Habib^TM^ EUS-RFA (EMcision Ltd., London, UK) probe (10- and 20-mm electrode) in locally advanced unresectable PDAC [31,32], reporting a 21% rate of mild AEs. Based on these promising reports, further studies and randomized trials are required to optimize the ablation parameters, still not standardized, and to prove whether the OS of these patients can be further improved by LTA and combined CT. Using the most widely applied EUS-guided RFA with different probe’s tip length in multi-centre studies could overcome the problem of slow enrolment we have had in this phase II RCT.

The rate and the type of AEs following EUS-HTP (29.7%) is similar to those reported from the above-mentioned studies, with no severe AEs and pancreatitis, confirming the safety of LTA under EUS-guidance in PDAC. To date, only two cases of pancreatitis, treated conservatively, were reported in the two available case series treating small non-functional pancreatic neuroendocrine tumours by EUS-guided RFA [6]. 

We observed at 6-months a 11.2% (Choi criteria) higher percentage of patients still being PD-free in the HTP-CT arm over the CT arm, albeit without a significant difference. According to the original phase II trial design, with a difference of 5–19% in the 6-PFS rate between the two arms the trial could be continued with a deemed sample size recalculation. Based on the observed 6-PFS rates in this phase II RCT, with 0.05 type I and 0.20 type II errors, the deemed sample size to reach ≥20% improved 6-PFS rate adding HTP to CT would be of 288 patients per arm. This accrual was unfortunately unfeasible, so the study was stopped due to the study protocol’s high demand to the centres.

With respect to the CT arm, we found in the HTP-CT arm a 10.7% and 17.2% higher rate of biological response and vital tumour volume reduction up to 6-months, along with a 21.6% higher median vital tumour volume reduction rate compared with baseline.

Recently, circulating CA19.9 and tumour volume changes have been proposed as complementary measures to the radiological response, which can be troublesome in PDAC, for a more accurate assessment of the treatment’s activity [13,16,17].

The 6-months results from this phase II trial may suggest that EUS-HTP added to CT in LA/BR-PDAC leads to a better tumour local control than CT alone, due to tumour cytoreductive effect [33].

However, EUS-HTP did not impact the resection rate, that was 8.2% lower in the HTP-CT arm than CT arm, although not significantly different, as well as the median PFS-time and OS-time.

In the HTP-CT arm, data on PFS and OS outcomes had a wide range (2–40 and 5–45 months, respectively), reflecting the different response to EUS-HTP for each patient. Although we enrolled a homogeneous set of consecutive patients with LA/BR-PDAC, 3 patients of the HTP-CT arm and 4 patients of the CT arm showed 2-months distant PD, likely suggesting pre-existing undetectable micro-metastasis. Excluding these patients, the median OS-time was 2-months longer for the HTP-CT arm than CT arm, in contrast with the longer OS reported in the CT arm than HTP-CT arm including all patients.

Moreover, although not significantly, baseline tumour size and volume were larger (about 11% and 58%, respectively) in the HTP-CT arm than CT arm. This difference might also explain the lower resection rate and similar PFS-time and OS-time following HTP-CT compared to CT. In a recent meta-analysis, PDAC tumour size showed a major impact on OS, with an increase of the death rate of about 4% with each cm-increase [34].

It is likely that a better patients’ selection also using molecular biomarkers predicting local but not distant PD may help improving the LTA efficacy. Recently, SMAD4 status was, indeed, reported as the only independent predictor of survival (*p* = 0.05) after intra-operative RFA [35].

Few studies investigated the long-term outcomes following LTA in PDAC. Regarding the survival, this RCT is not comparable to other studies, mainly because ablation was given as upfront therapy.

Previous studies investigated selected small groups of patients with short follow-up. Those non-randomized studies showed promising OS up to 25.6 and 16.2 months after intra-operative RFA and cryosurgery in LA-PDAC pre-treated with CT. A retrospective comparative study between CT and CT plus RFA reported a mean OS rising from 13 months for CT to 20 months for the combined approach. Another retrospective study found that the median OS after intra-operative RFA as initial treatment was 14.7 months versus 25.6 months with RFA as secondary treatment. In these patients, the median OS increased to 34 months using a combined triple approach including RFA, radio-chemotherapy and intra-arterial plus systemic CT [25,36].

The positioning and timing of LTA remain matters of debate. To date, no prospective comparisons were made between upfront LTA combined with CT or performed following induction CT strategies.

On the other hand, as PDAC is also insensitive to radiotherapy and often to CT, it has recently been shown that LTA may prime a systemic anti-tumour immune response in PDAC, possibly activated by the exposition to tumour-specific antigens released by the in-situ necrotic tissue [33,37,38]. The recent finding of a longer median OS in metastatic PDAC patients treated with cryo-immunotherapy or cryotherapy than those treated with immunotherapy and CT alone may support this hypothesis [39].

The cost-effectiveness of EUS-guided LTA of pancreatic cancer represents another interesting point to face, since there are no studies on this topic. The present study was not aimed at investigating this outcome and the applied probe was not commercially available, making this calculation difficult. The increased costs of an EUS-guided LTA, at any rate, include the device, the treatment and the hospitalization.

## 5. Conclusions

In conclusion, this phase II RCT was not adequately powered for the primary endpoint. Moreover, in such a small study the basal tumour volume’s and serum CA19.9 level’s difference, even if not significantly, as well as the heterogeneous CT regimens that were employed may represent additional limitations adding to the study heterogeneity and affecting results. On the other hand, we believe that this makes the results readily usable in a real-life setting.

Thus, the observed marginal 6-PFS benefit in favour of EUS-LTA should be interpreted with caution. Since no final PFS-time and OS-time benefit was observed adding EUS-LTA to CT, as PDAC is a systemic disease, we could hypothesize that EUS-LTA may be a safe adjunct for local disease control in carefully selected non-PD patients after induction CT. More efforts are needed to select patients who could benefit most from LTA, possibly combined with novel targeted agents or immunotherapy.

## Figures and Tables

**Table 1:** Baseline clinical and tumour features of the enrolled patients according with randomization in the two treatment arms.

Variables	HTP-CT Arm	CT Arm	*p*-Value
Patients enrolled, N.	17	20	
Sex, males/females, N. (%)	9 (52.9)/8 (47.1)	9 (45)/11 (55)	0.74
Age (years), mean ± StDe	63.5 ± 11.4	65.1 ± 5.03	0.58
Tumour site, head/body-tail, N. (%)	10 (58.8)/7(41.2)	12 (60)/8 (40)	1.00
Tumour size (mm) at MDCT scan, mean (± StDe)			
short axis	33.3 ± 9	29.8 ± 10.7	0.51
long axis	47.1 ± 11.6	43 ± 16.2	0.30
Tumour volume (cc) at MDCT scan, median (IQR)	32.1 (22.8–47.5)	20.2 (11–28.4)	0.23
Tumour staging, N. (%)			
borderline resectable	5 (29.4)	5 (25)	1.00
locally advanced	12 (70.6)	15 (75)	
7th TNM classification, N. (%) [11]			
T3	6 (35.3)	9 (45)	0.55
T4	7 (41.2)	6 (30)	0.48
8th TNM classification, N. (%) [11]			
T2	1 (5.9)	1 (5)	0.91
T3	0	2 (10)	0.19
T4	3 (17.6)	2 (10)	0.51
CA19.9 serum levels (U/mL), median (IQR)	733.2 (54.4–4049.9)	835 (75.2–5411.2)	0.61
>upper laboratory normal limit, N. (%)	16 (94.1)	14 (70)	
Metal biliary stent, N. (%)	8 (47.05)	10 (50)	0.88
Vessel invasion in locally advanced, N. (%) *			
superior mesenteric/portal vein	10 (83.3)	13 (86.7)	
hepatic artery	2 (16.7)	3 (20)	
superior mesenteric artery	7 (58.3)	5 (33.3)	
celiac axis	2 (16.7)	3 (20)	
splenic vein	3 (25)	5 (33.3)	
splenic artery	5 (41.7)	4 (26.7)	
gastroduodenal artery	0	3 (20)	
Vessel invasion in borderline resectable, N. (%) *			
superior mesenteric/portal vein	4 (80)	5 (100)	
splenic vein	1 (20)	0	
splenic artery	1 (20)	0	

HTP-CT: HybridTherm Probe plus Chemotherapy; CT: Chemotherapy; N: number; cc: ce timeter cube; StDe: standard deviation; IQR: interquartile range; MDCT: Multi-detector computed tomography; TNM: tumour-node-metastasis. * Percentages added to more than 100% because some patients having more than one vessel invaded, thus no statistical comparison was made for variables related to vessel invasion.

**Table 2:** Rates of radiological disease control (partial response + stable disease) at 2-, 4- and 6-months restaging, according Table 1. Patients experiencing progression disease at a restaging time-point were excluded from the analysis of the radiological response at the subsequent restaging time-point.

6-Months Progression-Free Survival and Radiological Response to Therapy	HTP-CT Arm		CT Arm		*p*-Value	
	*PP-Set*	*ITT-Set*	*PP-Set*	*ITT-Set*	*PP-Set*	*ITT-Set*
*Radiological disease control rate (Choi criteria)*						
2 months, % (N.)	80 (12/15)	71 (12/17)	72.2 (13/18 *)	65 (13/20)	0.61	0.70
	N. 8 PR, N. 4 SD		N. 7 PR, N. 6 SD			
4 months, % (N.)	66.7 (8/12)	47.1 (8/17)	69.2 (9/13 ^§^)	45 (9/20)	0.90	0.90
	N. 3 PR, N. 5 SD		N. 5 PR, N. 4 SD			
6 months, % (N.)	87.5 (7/8)	41.2 (7/17)	66.7 (6/9)	30 (6/20)	0.33	0.48
	N. 4 PR, N. 3 SD		N. 3 PR, N. 3 SD			
Overall response to therapy, % (N.)	80 (12/15)	71 (12/17)	72.2 (13/18 *)	65 (13/20)	0.88	0.95
	N. 9 PR, N. 3 SD		N. 11 PR, N. 3 SD			
6-PFS, % (N.)	46.7 (7/15)	41.2 (7/17)	35.3 (6/17)	30 (6/20)	0.52	0.48
*Radiological disease control rate (RECIST1.1)*						
2 months, % (N.)	80 (12/15)	71 (12/17)	83.3 (15/18)	75 (15/20)	0.81	0.79
	N. 12 SD		N. 4 PR, N. 11 SD			
4 months, % (N.)	75 (9/12)	52.9 (9/17)	71.4 (10/14 ^§^)	50 (10/20)	0.84	0.86
	N. 9 SD		N. 1 PR, N. 9 SD			
6 months, % (N.)	100 (9/9)	52.9 (9/17)	70 (7/10)	35 (7/20)	0.08	0.28
	N. 2 PR, N. 7 SD		N. 1 PR, N. 6 SD			
Overall response to therapy, % (N.)	80 (12/15)	71 (12/17)	83.3 (15/18)	75 (15/20)	0.81	0.79
	N. 2 PR, N. 10 SD		N. 6 PR, N. 9 SD			
6-PFS, % (N.)	60 (9/15)	52.9 (9/17)	41.2 (7/17)	35 (7/20)	0.30	0.28

HTP-CT: HybridTherm Probe plus Chemotherapy; CT: chemotherapy; N: number; PR: partial response; SD: stable disease; PD: progression disease; PP: Per-Protocol; ITT: Intention-To-Treat; RECIST1.1: Response Evaluation Criteria in Solid Tumours, version 1.1; 6-PFS: 6-months progression-free survival. * One patient of the CT arm was lost to follow-up before 2-months restaging. ^§^ One patient of the CT arm was lost to follow-up after 2-months restaging.

**Table 3:** Rates of <50% (non-biochemical responders), 50–89% (minor biochemical responders) and ≥90% (major biochemical responders) decrease of CA19.9 serum levels (UI/mL) at 2-, 4- and 6-months follow-up. Only patients with basal upper limit of laboratory normal for CA19.9 were considered (HTP-CT arm: *n* = 16; CT arm: *n* = 14).

CA19.9 Percentage Change versus Basal Value	HTP-CT Arm		CT Arm		*p*-Value	
	*PP-Set*	*ITT-Set*	*PP-Set*	*ITT-Set*	*PP-Set*	*ITT-Set*
CA19.9 decrease < 50%, % (N.)						
2 months	0	0	21.4 (3/14 *)	21.4 (3/14)	0.07	0.06
4 months	0	0	15.4 (2/13)	14.3 (2/14)	0.15	0.12
6 months	0	0	18.2 (2/11)	14.3 (2/14)	0.15	0.12
CA19.9 decrease 50–89%, % (N.)						
2 months	71.4 (10/14)	62.5 (10/16)	50 (7/14 *)	50 (7/14)	0.26	0.50
4 months	61.5 (8/13)	50 (8/16)	38.5 (5/13)	35.7 (5/14)	0.25	0.44
6 months	54.5 (6/11)	37.5 (6/16)	18.2 (2/11)	14.3 (2/14)	0.08	0.16
CA19.9 decrease ≥ 90%, % (N.)						
2 months	14.3 (2/14)	12.5 (2/16)	21.4 (3/14 *)	21.4 (3/14)	0.63	0.52
4 months	23.1 (3/13)	18.8 (3/16)	30.8 (4/13)	28.6 (4/14)	0.66	0.53
6 months	27.3 (3/11)	18.8 (3/16)	36.4 (4/11)	28.6 (4/14)	0.65	0.53
Overall CA19.9 decrease ≥ 50%, % (N.)	85.7 (12/14)	75 (12/16)	64.3 (9/14)	64.3 (9/14)	0.20	0.53

HTP-CT: HybridTherm Probe plus Chemotherapy; CT: chemotherapy; N: number; PP: Per-Protocol; ITT: Intention-To-Treat. * One patient of the CT arm was lost to follow-up after 2-months restaging.

**Table 4:** Median (IQR) basal and post-treatment tumour volume at 2-months (HTP-CT arm *n* = 14; CT arm *n* = 17), 4-months (HTP-CT arm *n* = 12; CT arm *n* = 16) and 6-months (HTP-CT arm: *n* = 10; CT arm: *n* = 12) follow-up and median (IQR) vital tumour volume reduction rate versus baseline.

Vital Tumour Volume (cc)	HTP-CT Arm	CT Arm	*p*-Value	Reduction Rate vs. Basal Tumour Volume (Median, IQR)		*p*-Value vs. Basal Tumour Volume	
Basal, median, (IQR)	31.3	20.2	0.06	**HTP-CT Arm**	**CT Arm**	**HTP-CT Arm**	**CT Arm**
	(22.6–49.1)	(11–28.4)					
2 months, median (IQR)	30.4	18.1	0.02	−16.7	−5.9%	0.82	0.85
	(18.5–37)	(15.5–25.8)		(−38.5–45.1%)	(−21.6–3%)		
4 months, median (IQR)	31.4	16.7	0.17	−14.3	−20.5%	0.60	0.77
	(15.6–39.4)	(13.8–23)		(−64.6–51.1%)	(−28.3–9.6)		
6 months, median (IQR)	20.1	18.2	0.39	−43.7%	−22.1%	0.31	0.89
	(16.5–34.7)	(14.3–28.7)		(−64.8–27.7%)	(−31–43.9%)		

HTP-CT: HybridTherm Probe plus Chemotherapy; CT: Chemotherapy; TV: tumour volume; cc: centimeter cube; IQR: interquartile range; CI: confidence interval.

## Data Availability

The data presented in this study are available on request from the corresponding author. The data are not publicly available due to privacy restrictions.

## Supplementary Materials

The following are available online at https://www.mdpi.com/article/10.3390/cancers13184512/s1. Figure S1: Flow-chart of the study design. Figure S2: Median overall survival (OS) time (95% confidence interval) and hazard ratio (HR, 95% confidence interval) for death in patients treated with endoscopic ultrasound-guided ablation with HybridTherm Probe plus chemotherapy (HTP-CT arm) versus chemotherapy alone (CT arm): (A) Per-Protocol analysis (PP-set): 13 (8.8–25.6) versus 17 (10.9–56.7) months; HR HTP-CT arm/CT arm for death = 1.18 (0.6–2.4) (Log-rank test: Chi-squared = 0.20, *p* = 0.79); (B) Intention-To-Treat analysis (ITT-set): 13 (8.8–25.6) versus 17 (10.9–56.7) months; HR HTP-CT arm/CT arm for death = 1.18 (0.6–2.4) (Log-rank test: Chi-squared = 0.20, *p* = 0.79). Table S1: Chemotherapy regimens, dosages and administration timing. Table S2: Chemotherapy-related features of the patients’ cohort. Table S3: Pathological outcomes on surgical specimens in resected patients. Table S4: Chemotherapy-related grade III/IV adverse events, defined according to the National Cancer Institute’s Common Terminology Criteria for Adverse Events (version 4.0). The worst toxicity grade in every cycle for each adverse event type was considered.
