# Supplementary material for: Efficacy of Endoscopic Ultrasound-Guided Ablation with the HybridTherm Probe in Locally Advanced or Borderline Resectable Pancreatic Cancer: A Phase II Randomized Controlled Trial
Source: Cancers (Basel). 2021 Sep 8;13(18):4512. doi: 10.3390/cancers13184512 (PMC8464946; doi:10.3390/cancers13184512)
Supplement: Supplementary file 1 [file cancers-13-04512-s001.zip › cancers-1344460-supplementary.pdf]

cancers

Article

Efficacy of Endoscopic Ultrasound-Guided Ablation with the HybridTherm Probe in Locally Advanced or Borderline Resectable Pancreatic Cancer: A Phase II Randomized Controlled Trial

Sabrina Gloria Giulia Testoni, Maria Chiara Petrone, Michele Reni, Gemma Rossi, Maurizio Barbera, Valeria Nicoletti, Simone Gusmini, Gianpaolo Balzano, Walter Linzenbold, Markus Enderle, Emanuel Della-Torre, Francesco De Cobelli, Claudio Doglioni, Massimo Falconi, Gabriele Capurso and Paolo Giorgio Arcidiacono

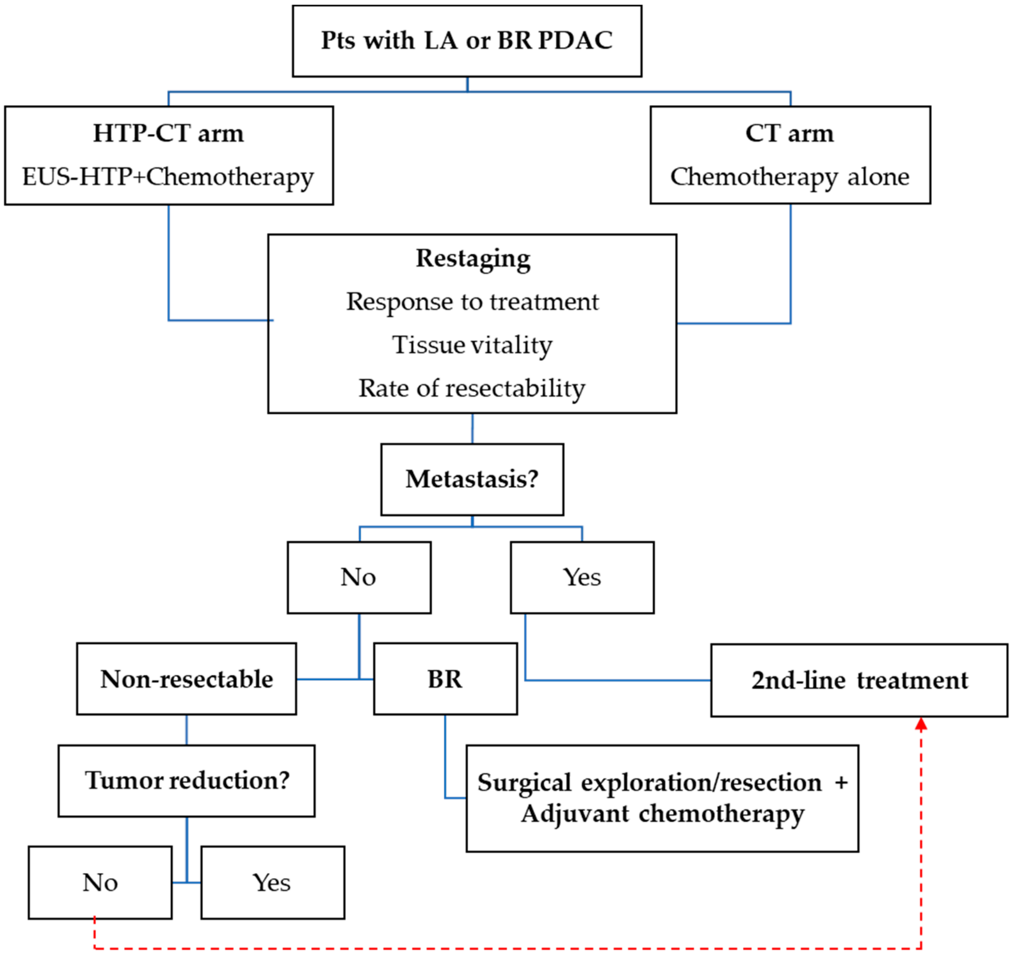

Figure S1. Flow-chart of the study design. LA: Locally advanced; BR: Borderline resectable; PDAC: pancreatic ductal adenocarcinoma; HTP-CT: HybridTherm Probe plus Chemotherapy; EUS-HTP: endoscopic ultrasound-guided ablation with HybridTherm Probe; CT: Chemotherapy.

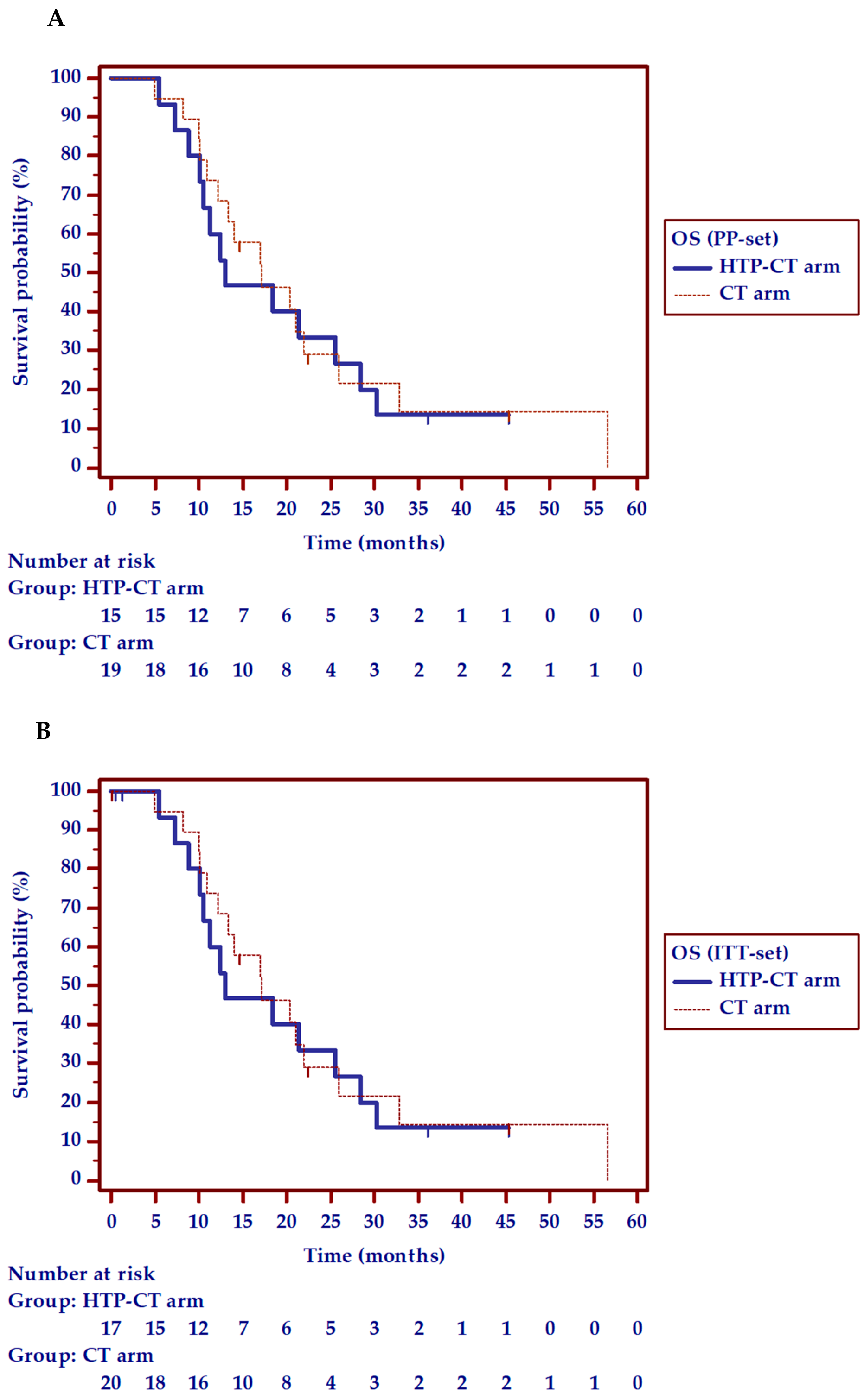

Figure S2: Median overall survival (OS) time (95% confidence interval) and hazard ratio (HR, 95% confidence interval) for death in patients treated with endoscopic ultrasound-guided ablation with HybridTherm Probe plus chemotherapy (HTP-CT arm) versus chemotherapy alone (CT arm): A) Per-Protocol analysis (PP-set): 13 (8.8 - 25.6) versus 17 (10.9 - 56.7) months; HR HTP-CT arm/CT arm for death = 1.18 (0.6 - 2.4) (Log-rank test: Chi-squared = 0.20, p = 0.79); B) Intention-To-Treat analysis (ITT-set): 13 (8.8 – 25.6) versus 17 (10.9 – 56.7) months; HR HTP-CT arm/CT arm for death = 1.18 (0.6 – 2.4) (Log-rank test: Chi-squared = 0.20, p = 0.79). Short vertical lines represent censured patients.

Table S1. Chemotherapy regimens, dosages and administration timing.

CT regimens (AIOM)	Drugs	Dosage and administration timing
PEXG	Cisplatin	30 mg/m^2 on days 1 and 15
	Epirubicin	30 mg/m^2 on days 1 and 15
	Gemcitabine	800 mg/m^2 on days 1 and 15
	Capecitabine	1250 mg/m^2 on days 1 to 28
Gemcitabine	Gemcitabine	1000 mg/m^2 on days 1, 8, 15
FOLFIRINOX	Oxaliplatin	85 mg/m^2 on days 1 and 15
	Leucovorin	400 mg/m^2 on days 1 and 15
	Irinotecan	180 mg/m^2 on days 1 and 15
	Fluorouracil	400 mg/m^2 bolus followed by 2400 mg/m2 on days 1 and 15
Nab-Paclitaxel + Gemcitabine	Nab-paclitaxel	125 mg/m2 on days 1, 8, 15
	Gemcitabine	1000 mg/m2 on days 1, 8, 15

AIOM: Italian Association of Medical Oncology; CT: Chemotherapy.

Table S2. Chemotherapy-related features of the patients' cohort.

Variables	HTP-CT arm	CT arm	p-value
Time between PDAC diagnosis and start of CT (days), mean ± StDe	37.7 (± 11.7)	32.8 (± 13.3)	0.27
Time between randomization and start of CT (days), mean ± StDe	24.7 (± 12.2)	22.2 (± 13.9)	0.58
CT regimen, N. (%)			
Gemcitabine + Nab-Paclitaxel	13 (76.5)	13 (65)	0.45
Folfirinox	2 (11.8)	5 (25)	0.31
Gemcitabine	0	1 (5)	0.36
CT duration (days), mean ± StDe	142.9 (± 49)	154.8 (± 56.2)	0.53
6-months completed CT, N. (%)	12 (80)	12 (70.6)	0.55
CT interruption before 6-months, N. (%)	4 (26.7)	5 (29.4)	0.87
Haematological toxicity, N.	1	0	
Extra-haematological toxicity, N.	0	2	
Progression disease, N.	2	2	
Surgery before the end of 6-months CT, N.	0	1	
Physician's choice, N.	1	0	
Chemo-radiotherapy after induction CT, N (%)	10 (66.7)	11 (64.7)	0.90

PDAC: pancreatic ductal adenocarcinoma; HTP-CT: HybridTherm Probe plus Chemotherapy; CT: Chemotherapy; StDe: standard deviation; N: number.

Table S3. Pathological outcomes on surgical specimens in resected patients.

Variables	HTP-CT arm	CT arm
Resected patients % (N)		
PP-set	2/15 (13.3)	4/17 (23.5)
ITT-set	2/17 (11.8)	4/20 (20)
R status % (N)	2/2 (100)	3/4 (75)
R0 resection rate	0	1/4 (25)
Type of resection, N (%)		
Pancreaticoduodenectomy	1/2 (50)	3/4 (75)
Distal pancreatectomy	1/2 (50)	0
Total pancreatectomy	0	1/4 (25)
TNM 7th classification [11]		
T code, N (%)		
pT2	-	-
pT3	1/2 (50)	1/4 (25)
N status, N (%)		
pN0	-	1/4 (25)
pN1	1/2 (50)	-
pN2	-	-
Grade, N (%)		
G2	1/2 (50)	1/4 (25)
G3	-	-
TNM 8th classification [11]		
T code, N (%)		
pT2	1/2 (50)	2/4 (50)
pT3	-	1/4 (25)
N status, N (%)		
pN0	1/2 (50)	-
pN1	-	1/4 (25)
pN2	-	2/4 (50)
Grade, N (%)		
G2	-	1/4 (25)
G3	1/2 (50)	2/4 (50)
Pathological response (Hartman et al.) [23], N (%)		
Marked-Complete	0	1/4 (25)
Minimum-Moderate	2/2 (100)	1/4 (25)
Poor	0	2/4 (50)

HTP-CT: HybridTherm Probe plus Chemotherapy; CT: Chemotherapy; N: number; PP: per-protocol; ITT: intention-to-treat; TNM: tumour-node-metastasis.

Table S4. Chemotherapy-related grade III/IV adverse events, defined according to the National Cancer Institute's Common Terminology Criteria for Adverse Events (version 4.0). The worst toxicity grade in every cycle for each adverse event type was considered.

Reasons for chemotherapy interruption	HTP-CT arm		CT arm		p-value	
	PP-set	ITT-set	PP-set	ITT-set	PP-set	ITT-set
Anemia, N (%)						
Grade III	3/15 (20)	3/17 (17.6)	2/17 (11.8)	2/20 (10)	0.53	0.51
Grade IV	0	0	0	0		
Neutropenia, N (%)						
Grade III	2/15 (13.3)	2/17 (11.8)	3/17 (17.6)	3/20 (15)	0.74	0.78
Grade IV	1/15 (6.7)	1/17 (5.9)	3/17 (17.6)	3/20 (15)	0.36	0.38
Thrombocytopenia, N (%)						
Grade III	1/15 (6.7)	1/17 (5.9)	1/17 (5.9)	1/20 (5)	0.93	0.90
Grade IV	1/15 (6.7)	1/17 (5.9)	0	0	0.28	0.28
Nausea, N (%)						
Grade III	1/15 (6.7)	1/17 (5.9)	3/17 (17.6)	3/20 (15)	0.36	0.38
Grade IV	0	0	0	0		
Diarrhea, N (%)						
Grade III	1 /15 (6.7)	1/17 (5.9)	1/17 (5.9)	1/20 (5)	0.93	0.90
Grade IV	0	0	0	0		
Mucositis, N (%)						
Grade III	1/15 (6.7)	1/17 (5.9)	0	0	0.28	0.28
Grade IV	0	0	0	0		
Fatigue, N (%)						
Grade III	0	0	3/17 (17.6)	3/20 (15)	0.09	0.10
Grade IV	0	0	0	0		
Neuropathy, N (%)						
Grade III	2/15 (13.3)	2/17 (11.8)	4/17 (23.5)	4/20 (20)	0.47	0.51
Grade IV	0	0	0	0		
Fever, N (%)						
Grade III	2/15 (13.3)	2/17 (11.8)	2/17 (11.8)	2/20 (10)	0.90	0.86
Grade IV	0	0	0	0		
Cholangitis, N (%)						
Grade III	1/15 (6.7)	1/17 (5.9)	1/17 (5.9)	1/20 (5)	0.93	0.90
Grade IV	0	0	0	0		

HTP-CT: HybridTherm Probe plus Chemotherapy; CT: Chemotherapy; N: number.
